# Diagnostic Utility of Immunohistochemistry in Lung Carcinoma Subtyping: A Clinicopathological Study From a Tertiary Care Center

**DOI:** 10.7759/cureus.104891

**Published:** 2026-03-09

**Authors:** Aishwarya Mantri, Devanshi Pathak, Matariswa Samanta, Jay Amit Haripura, Pawan Nikhra

**Affiliations:** 1 Pathology, Pacific Institute of Medical Sciences, Sai Tirupati University, Udaipur, IND

**Keywords:** diagnostic accuracy, immunohistochemistry, lung carcinoma, p40, ttf-1

## Abstract

Introduction: Accurate subtyping of lung carcinomas on small biopsies is critical for targeted therapy selection; however, regional data on the diagnostic performance of immunohistochemical (IHC) markers remain limited. This study aimed to evaluate the histomorphological spectrum and diagnostic utility of a comprehensive IHC panel for lung carcinoma.

Materials and methods: A retrospective analysis was conducted on 97 lung biopsy cases collected between January 2024 and December 2025 at a tertiary care center in Rajasthan, India. Formalin-fixed paraffin-embedded tissues were evaluated using hematoxylin and eosin (H&E) staining and IHC markers, including p40, p63, thyroid transcription factor-1 (TTF-1), Napsin-A, synaptophysin, chromogranin, WT-1, calretinin, and pancytokeratin. Tumors were subclassified based on combined morphologic and IHC findings.

Results: Adenocarcinoma (56, 57.7%) and squamous cell carcinoma (33, 34.0%) were the predominant subtypes. Most patients were male (76, 78.4%) and in the 61-70 years age group (42, 43.3%). Bronchoscopy-guided biopsies (72, 74.2%) were the most common, with the right lower lobe as the most frequent site (31, 32.0%). For squamous cell carcinoma, p40 demonstrated an overall diagnostic accuracy of 96.7% with 100% specificity. For adenocarcinoma, TTF-1 showed 95.1% accuracy with 96.0% sensitivity, while Napsin-A showed 93.8% accuracy with 100% specificity. Neuroendocrine markers (synaptophysin and chromogranin) and mesothelial markers (WT-1 and calretinin) demonstrated 100% diagnostic performance metrics in their respective categories.

Conclusion: A morphology-guided IHC approach using a limited panel of markers, including TTF-1 and Napsin-A for adenocarcinoma and p40 with or without p63 for squamous cell carcinoma, demonstrated high diagnostic accuracy in the subclassification of lung carcinomas on biopsy specimens. These findings are consistent with current WHO recommendations and support the practical utility of focused IHC panels in routine diagnostic practice. Such strategies may help achieve accurate tumor subtyping while facilitating efficient use of limited biopsy tissue.

## Introduction

Lung cancer is the leading cause of cancer-related morbidity and mortality worldwide and continues to pose a major global health challenge [1]. According to GLOBOCAN 2022, approximately 2.48 million new cases of lung cancer were diagnosed globally, accounting for 12.4% of all cancers, and it remains the foremost cause of cancer-related deaths [2]. In India, lung carcinoma constitutes one of the most commonly diagnosed malignancies in tertiary care centers, with a rising incidence attributed to tobacco consumption, environmental pollution, and increasing urbanization [3]. In 2022, the age-standardized incidence rate (ASIR) was 8.5 per 100,000 in men and 3.2 per 100,000 in women, while the age-standardized mortality rate (ASMR) was 7.8 per 100,000 in men and 2.9 per 100,000 in women in India [4].

Accurate histological diagnosis and subclassification of lung cancer are crucial for appropriate patient management and prognostication. Lung biopsy, particularly small core (true-cut) biopsies with limited tissue volume, remains a reliable diagnostic modality in routine practice. Histopathological evaluation, supplemented by immunohistochemistry (IHC), allows precise classification of primary lung malignancies, which is particularly important because these small biopsy specimens often represent advanced disease with limited tissue and may carry a poorer prognosis if not accurately classified, especially in small or poorly differentiated tumor samples [5].

The 2021 World Health Organization (WHO) classification of thoracic tumors categorizes lung neoplasms primarily into adenocarcinoma, squamous cell carcinoma, neuroendocrine neoplasms (including small cell carcinoma and carcinoid tumors), and other less common epithelial and mesenchymal tumors, using an integrated approach based on histomorphological, immunohistochemical, and molecular features [6]. This classification is clinically significant, as contemporary treatment strategies, including targeted therapies and immunotherapy, are highly subtype-specific. Adenocarcinoma is frequently associated with actionable molecular alterations, whereas squamous cell carcinoma and small cell carcinoma follow distinct therapeutic pathways [7].

IHC has therefore become an indispensable adjunct in the diagnostic work-up of lung tumors by facilitating objective identification of tumor lineage and differentiation. Thyroid transcription factor-1 (TTF-1) and Napsin-A are commonly employed for the diagnosis of adenocarcinoma, while p40 and p63 serve as reliable markers for squamous cell carcinoma. Neuroendocrine differentiation is confirmed using synaptophysin and chromogranin, and mesothelial lineage is supported by WT-1 and calretinin. Pancytokeratin is useful in establishing epithelial differentiation in poorly differentiated malignancies [8].

According to the WHO 2021 guidelines for the evaluation of small biopsies, a limited immunohistochemical panel comprising TTF-1 for adenocarcinoma and p40 for squamous cell carcinoma, along with mucin stains, is recommended for the subtyping of non-small cell lung carcinoma to conserve tissue for molecular testing [6]. Molecular testing for specific genetic mutations or biomarkers serves as an adjunct for more rational, targeted treatment regimens.

Despite the widespread use of these markers, regional data evaluating their diagnostic performance in routine practice remain limited. Moreover, variations in sensitivity and specificity across different studies highlight the need for institutional and population-based validation. The present study aimed to analyze the histomorphological spectrum of lung tumors and to evaluate the diagnostic utility of a panel of immunohistochemical markers, including TTF-1, Napsin-A, p63, and p40, for accurate subclassification of lung carcinoma.

## Materials and methods

This retrospective study was conducted in the Department of Pathology at Pacific Institute of Medical Sciences, Udaipur, Rajasthan, India, over a period of two years from January 2024 to December 2025. The study was undertaken after obtaining approval from the Institutional Ethics Committee (IEC approval number: STU/IEC/2026/034).

All lung biopsy specimens received during the study period with a histopathological diagnosis of lung carcinoma were included. Clinical details such as age, sex, site of lesion, radiological findings, type of biopsy, and provisional clinical diagnosis were retrieved from hospital records and pathology requisition forms. Cases with inadequate tissue, extensive necrosis, poor preservation, or incomplete clinical and pathological data were excluded from the study.

Formalin-fixed, paraffin-embedded tissue blocks of the included cases were retrieved from the archives. Sections of 3-4 µm thickness were cut and stained with hematoxylin and eosin (H&E) for histopathological evaluation. Tumors were classified according to the WHO 2021 classification of lung tumors based on histopathological slides [6].

IHC was performed on representative sections using a polymer-based horseradish peroxidase (HRP) detection system (Super Sensitive™ Polymer-HRP IHC Detection System, BioGenex Laboratories, Fremont, CA, USA). Tissue sections were first deparaffinized using X-Dewax™ tissue deparaffinization solution (Catalog No. HX016-XAK, Lot: HX0161025I, BioGenex), followed by rehydration through graded alcohols. Antigen retrieval was carried out using heat-induced epitope retrieval with the appropriate retrieval buffer as recommended by the manufacturer.

The primary antibody panel included p40, p63, TTF-1, Napsin-A, synaptophysin, chromogranin, WT-1, calretinin, and pancytokeratin. Sections were incubated with primary antibodies according to the manufacturer’s recommended protocol, followed by incubation with Super Enhancer reagent (Lot: HX0270525N, BioGenex) and Polymer-HRP detection reagent (HX030-20XN, BioGenex). Chromogenic visualization was performed using 3,3′-diaminobenzidine (DAB) chromogen with stable DAB buffer (Lot: HX0290525, BioGenex). Slides were counterstained with hematoxylin (BioGenex), dehydrated, and mounted. Appropriate positive and negative controls were included with each batch of staining [9].

Immunohistochemical staining was performed using an automated NanoVIP300 IHC stainer (BioGenex Laboratories). Stained slides were independently evaluated by pathologists and interpreted based on nuclear staining for p40, p63, and TTF-1; cytoplasmic staining for Napsin-A, synaptophysin, chromogranin, calretinin, and pancytokeratin; and nuclear or cytoplasmic staining for WT-1 where applicable [10]. A case was considered positive when at least 5% of tumor cells demonstrated moderate to strong specific staining [11]. Based on the combined histomorphological and immunohistochemical findings, tumors were categorized into adenocarcinoma, squamous cell carcinoma, small cell carcinoma, poorly differentiated carcinoma, mesothelioma, or metastatic carcinoma.

Statistical analysis was performed using SPSS version 26.0 (IBM Corp., Armonk, NY, USA). Data were expressed as frequencies and percentages. For each immunohistochemical marker, a 2 × 2 contingency table was constructed by comparing immunohistochemical results with the final histopathological diagnosis, which was taken as the reference standard. True positives (TP) were cases in which the marker was positive in tumors of the corresponding subtype, while true negatives (TN) were cases in which the marker was negative in tumors not belonging to that subtype. False positives (FP) were cases in which the marker was positive in non-corresponding tumor subtypes, and false negatives (FN) were cases in which the marker was negative in the corresponding tumor subtype.

Sensitivity was calculated as TP / (TP + FN) × 100 and represented the ability of the marker to correctly identify tumors of the target subtype. Specificity was calculated as TN / (TN + FP) × 100 and reflected the ability of the marker to correctly exclude non-target tumor subtypes. Positive predictive value (PPV) was calculated as TP / (TP + FP) × 100, while negative predictive value (NPV) was calculated as TN / (TN + FN) × 100. Diagnostic accuracy was calculated as (TP + TN) / (TP + TN + FP + FN) × 100, representing the overall proportion of correctly classified cases.

## Results

Among 97 cases, the majority were in the 61-70 years age group (42, 43.30%), followed by 51-60 years (36, 37.11%). Males predominated the study sample (76, 78.35%). Most biopsies were bronchoscopy-guided (72, 74.23%), with CT-guided (18, 18.56%) and USG-guided (7, 7.22%) biopsies forming the remainder. Lesions were most commonly located in the right lower lobe (31, 31.96%) and left upper lobe (26, 26.80%). Clinically, malignancy was suspected in 94 cases (96.91%), while tuberculosis was considered in three cases (3.09%) (Table 1).

**Table 1:** Clinicodemographic profile and biopsy characteristics of the cases. This table presents the distribution of cases according to age group, gender, type of biopsy, site of lesion, and clinical diagnosis. Data are expressed as frequency (n) and percentage (%). CT: computed tomography; USG: ultrasonography.

Category		Frequency	Percentage
Age	<40 years	6	6.19%
	41-50 years	9	9.28%
	51-60 years	36	37.11%
	61-70 years	42	43.30%
	>70 years	4	4.12%
Gender	Male	76	78.35%
	Female	21	21.65%
Type of biopsy	Bronchoscopy guided	72	74.23%
	CT guided	18	18.56%
	USG guided	7	7.22%
Site of lesion	Right upper lobe	23	23.71%
	Right middle lobe	9	9.28%
	Right lower lobe	31	31.96%
	Left upper lobe	26	26.80%
	Left lower lobe	8	8.25%
Clinical diagnosis	Malignancy	94	96.91%
	Tuberculosis	3	3.09%

Final diagnosis revealed adenocarcinoma as the most common tumor type (56, 57.73%), followed by squamous cell carcinoma (33, 34.02%). Poorly differentiated carcinoma accounted for three cases (3.09%), while mesothelioma and metastatic carcinoma were each observed in two cases (2.06%). Small cell carcinoma was rare, with only one case (1.03%) identified in the present series (Table 2).

**Table 2:** Distribution of final histopathological diagnosis. This table shows the frequency distribution of different histopathological subtypes of lung carcinoma. Data are presented as the number of cases (n) and the percentage (%).

Final diagnosis	Frequency	Percentage
Adenocarcinoma	56	57.73%
Squamous cell carcinoma	33	34.02%
Mesothelioma	2	2.06%
Metastatic carcinoma	2	2.06%
Small cell carcinoma	1	1.03%
Poorly differentiated carcinoma	3	3.09%

**Figure 1:**
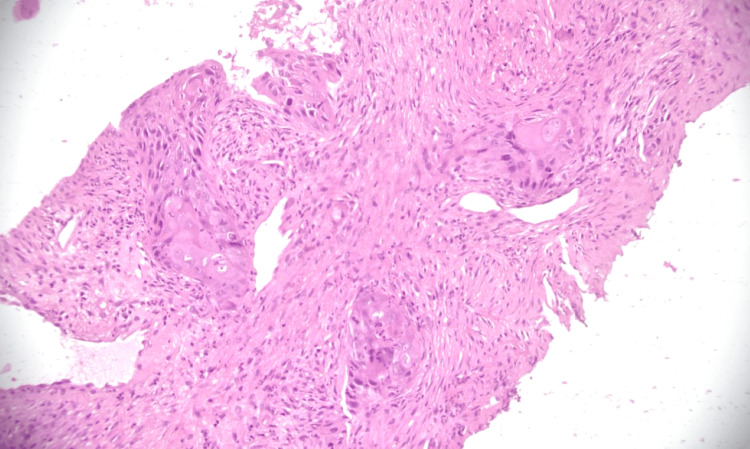
Histopathological features suggestive of squamous cell carcinoma (H&E, 200×). Photomicrograph of hematoxylin and eosin (H&E)-stained lung biopsy section showing tumor cells arranged in nests and sheets. Individual tumor cells display abundant eosinophilic cytoplasm and nuclear pleomorphism. Magnification: 200×.

**Figure 2:**
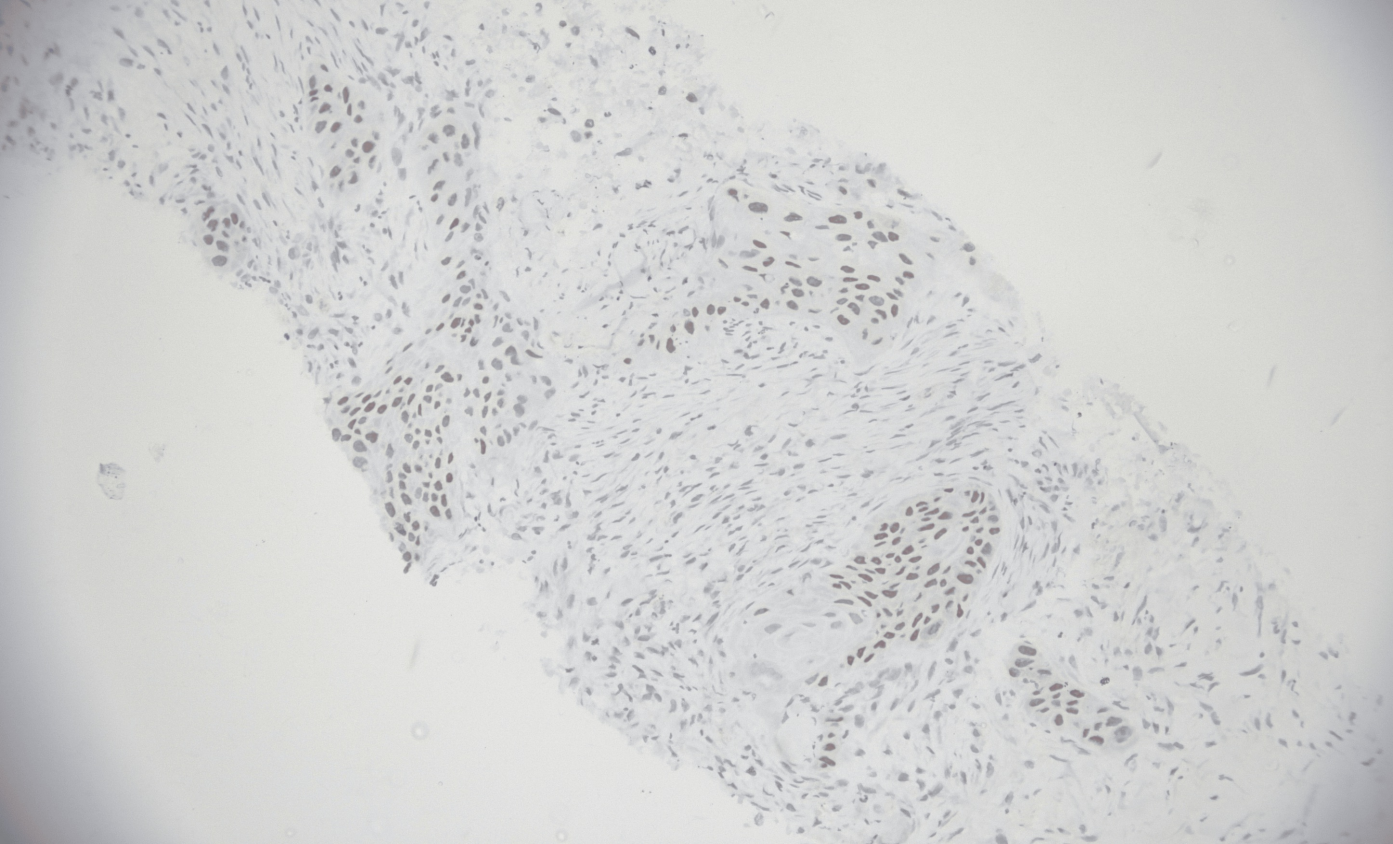
p40 immunohistochemical expression in squamous cell carcinoma (immunohistochemistry, 200×). Photomicrograph demonstrating strong nuclear immunoreactivity for p40 in tumor cells. Magnification: 200×.

**Figure 3:**
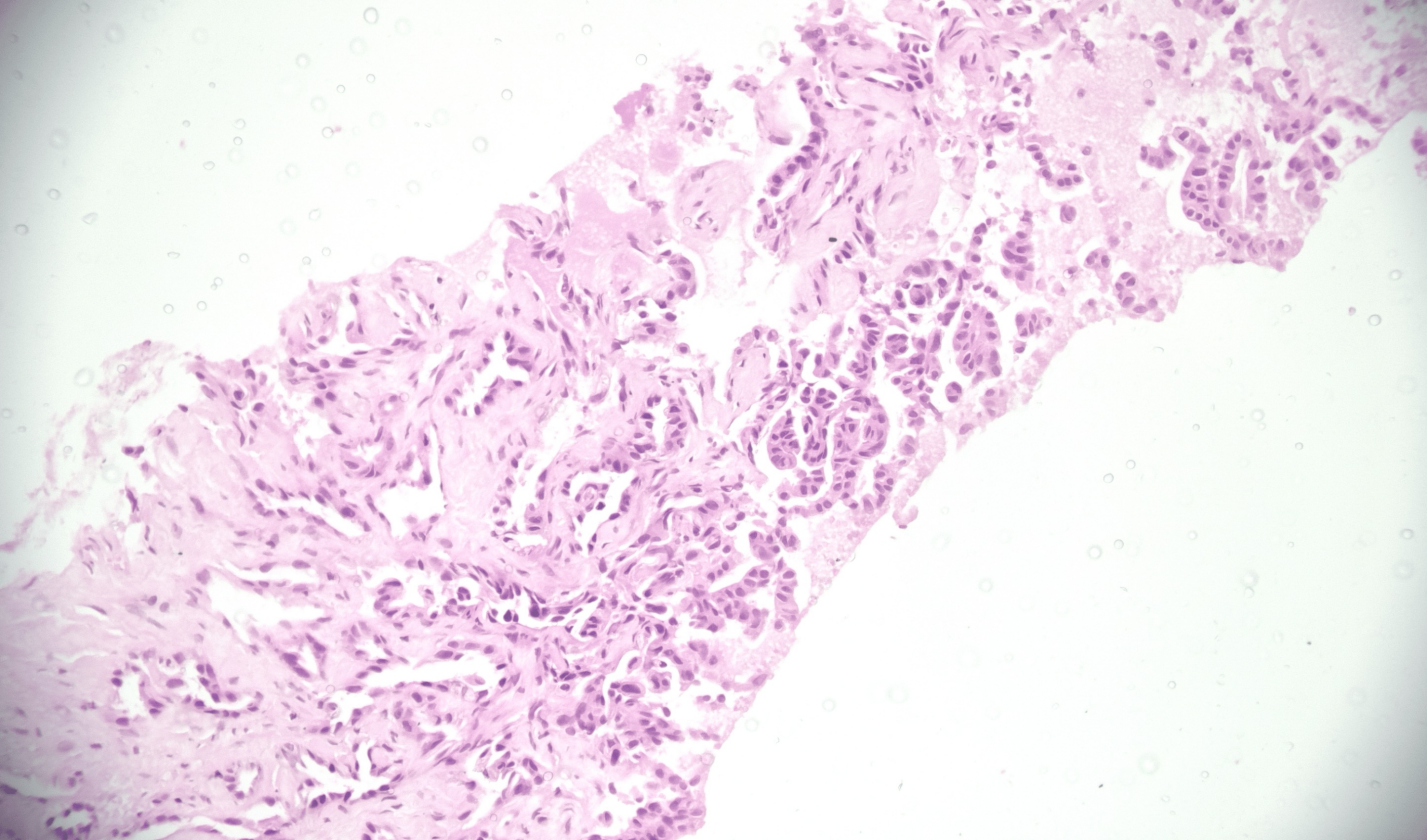
Histopathological features suggestive of adenocarcinoma (H&E, 200×). Photomicrograph showing tumor cells forming glandular structures with stromal invasion. Magnification: 200×.

**Figure 4:**
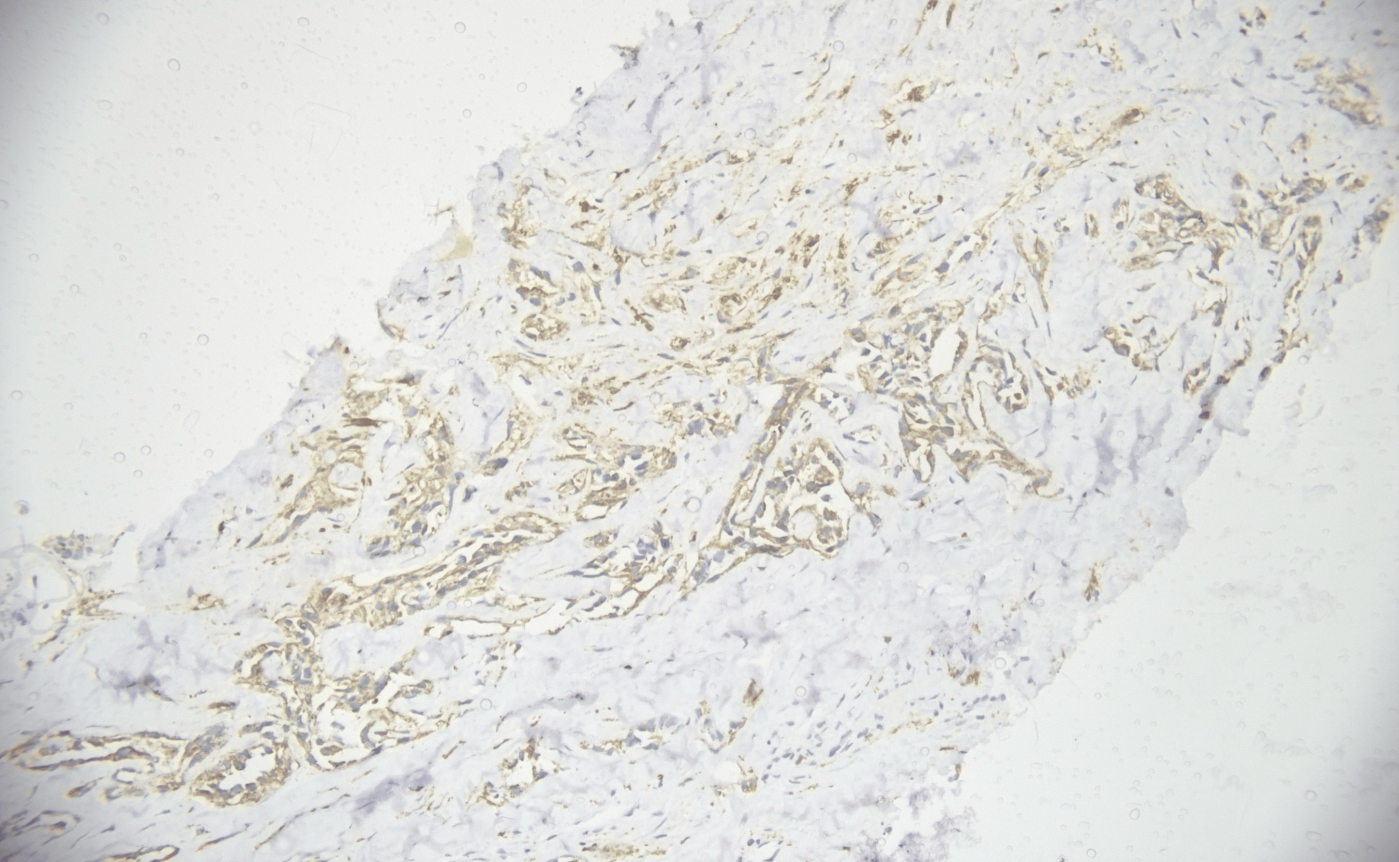
Napsin-A immunohistochemical expression in adenocarcinoma (immunohistochemistry, 200×). Photomicrograph demonstrating cytoplasmic immunoreactivity for Napsin-A in tumor cells. Magnification: 200×.

**Figure 5:**
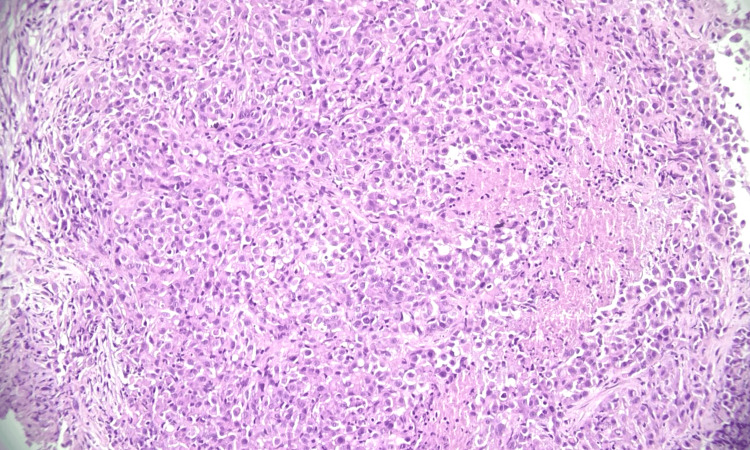
Histopathological features suggestive of mesothelioma (H&E, 200×). Photomicrograph of hematoxylin and eosin (H&E)-stained lung biopsy section showing tumor cells arranged in sheets with areas of background necrosis. Individual cells exhibit oval morphology with round nuclei and moderately eosinophilic cytoplasm. Magnification: 200×.

**Figure 6:**
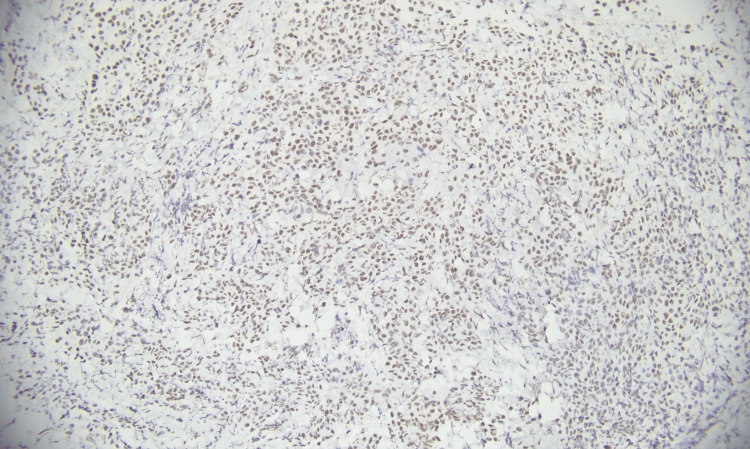
WT-1 immunohistochemical expression in mesothelioma (immunohistochemistry, 200×). Photomicrograph demonstrating nuclear immunoreactivity for WT-1 in tumor cells. Magnification: 200×.

**Figure 7:**
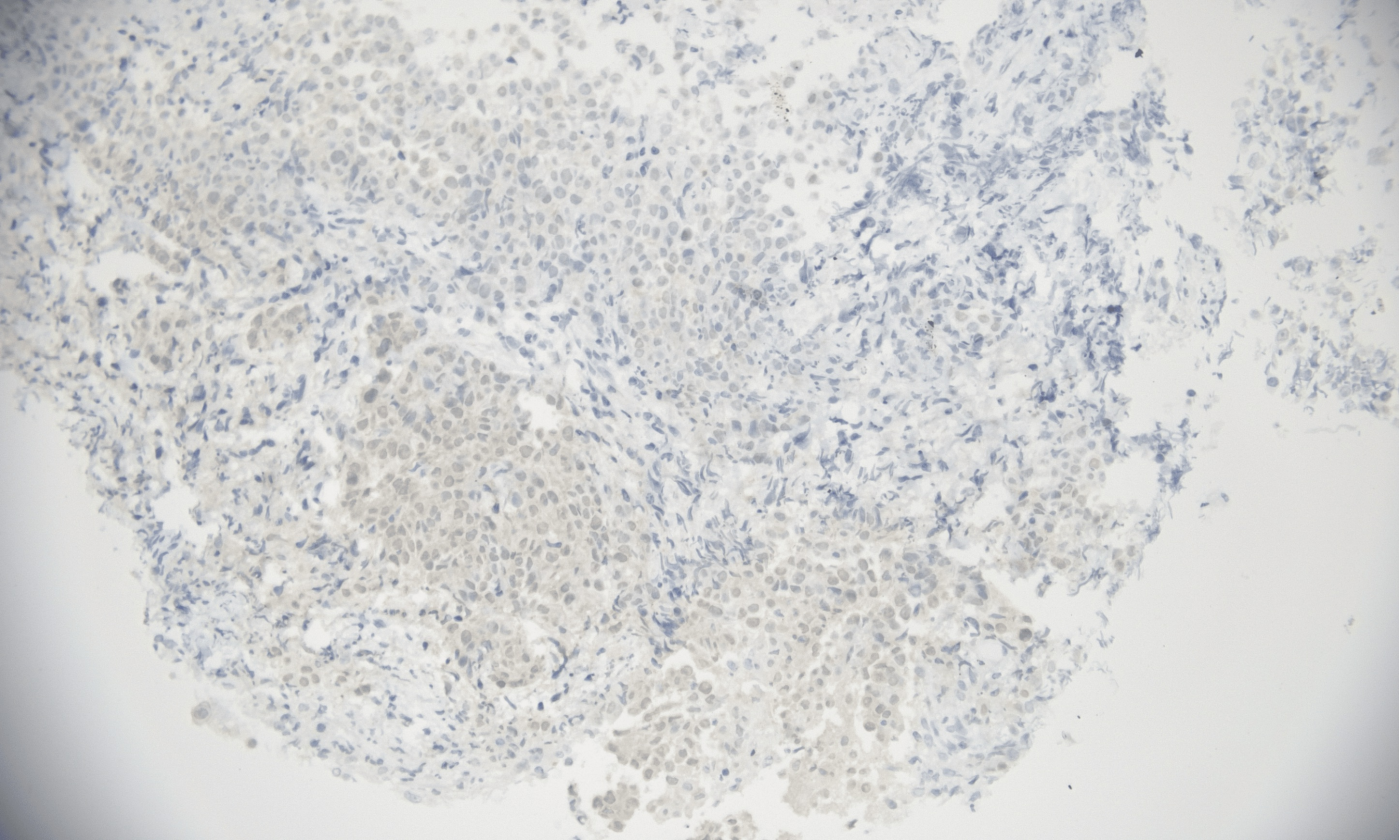
Calretinin immunohistochemical expression in mesothelioma (immunohistochemistry, 200×). Photomicrograph demonstrating cytoplasmic immunoreactivity for calretinin in tumor cells. Magnification: 200×.

P40 and P63 showed predominant expression in squamous cell carcinoma, with occasional positivity in adenocarcinoma and no reactivity in small cell carcinoma, poorly differentiated carcinoma, mesothelioma, or metastatic carcinoma. TTF-1 and Napsin-A demonstrated predominant expression in adenocarcinoma, with rare positivity in squamous cell carcinoma and consistent negativity in the remaining tumor categories. Neuroendocrine markers (synaptophysin and chromogranin) showed positivity exclusively in the case of small cell carcinoma. Mesothelial markers (WT-1 and calretinin) showed uniform positivity in mesothelioma and were negative in all other tumor types. Pan-cytokeratin demonstrated consistent positivity in poorly differentiated carcinoma and metastatic carcinoma, confirming epithelial differentiation in these tumors (Table 3 and Figures 1-7).

**Table 3:** Immunohistochemical marker expression across tumor types. This table depicts the expression profile of various immunohistochemical (IHC) markers across different histopathological tumor categories. Marker expression is presented as the number of positive cases over total cases tested (positive/total). TTF-1: thyroid transcription factor-1; WT-1: Wilms tumor-1; Pan-CK: pan-cytokeratin.

Marker	Adenocarcinoma (56/97)	Squamous cell carcinoma (33/97)	Small cell carcinoma (1/97)	Poorly differentiated carcinoma (3/97)	Mesothelioma (2/97)	Metastatic carcinoma (2/97)
P40	0/21	29/31	0/1	0/3	0/2	0/2
P63	1/13	23/25	0/1	0/3	0/2	0/2
TTF-1	48/50	2/23	0/1	0/3	0/2	0/2
Napsin-A	40/44	0/13	0/1	0/3	0/2	0/2
Synaptophysin	0/3	0/3	1/1	0/3	0/2	0/2
Chromogranin	0/3	0/3	1/1	0/3	0/2	0/2
WT-1	0/2	0/2	0/1	0/3	2/2	0/2
Calretinin	0/2	0/2	0/1	0/3	2/2	0/2
Pan-CK	1/4	0/4	0/1	3/3	0/2	2/2

Neuroendocrine markers (synaptophysin and chromogranin) and mesothelial markers (WT-1 and calretinin) demonstrated 100% sensitivity, specificity, positive predictive value, negative predictive value, and diagnostic accuracy within their respective tumor categories, although these findings were based on a limited number of cases. TTF-1 showed high sensitivity (96.0%) and specificity (93.5%), with an overall diagnostic accuracy of 95.1% for adenocarcinoma. P40 demonstrated high diagnostic accuracy (96.7%) for squamous cell carcinoma with 100% specificity, while P63 showed good sensitivity (92.0%) and specificity (95.2%) with a diagnostic accuracy of 93.5%. Napsin-A showed high specificity (100%) with balanced sensitivity (90.9%) and an overall diagnostic accuracy of 93.8% for adenocarcinoma (Table 4).

**Table 4:** Diagnostic performance of immunohistochemical markers. This table summarizes the diagnostic performance parameters of individual immunohistochemical markers, including sensitivity, specificity, positive predictive value (PPV), negative predictive value (NPV), and overall diagnostic accuracy.

Marker	Sensitivity (%)	Specificity (%)	PPV (%)	NPV (%)	Diagnostic accuracy (%)
P40	93.5	100	100	93.5	96.7
P63	92.0	95.2	95.8	90.9	93.5
TTF-1	96.0	93.5	96.0	93.5	95.1
Napsin-A	90.9	100	100	84.0	93.8
Synaptophysin	100	100	100	100	100
Chromogranin	100	100	100	100	100
WT-1	100	100	100	100	100
Calretinin	100	100	100	100	100

## Discussion

Advances in lung cancer therapy, particularly the expansion of targeted and subtype-directed treatment strategies, have substantially increased the importance of accurate histological classification at initial diagnosis [12]. As a large proportion of lung carcinomas present at an advanced and unresectable stage, diagnosis is frequently established on small bronchoscopic or image-guided core biopsies. In such settings, optimal utilization of limited tissue through careful morphologic evaluation supplemented by focused IHC is critical for precise tumor subclassification and for preserving material for downstream molecular testing. The findings of the present study indicate that a morphology-guided, panel-based IHC approach can achieve high diagnostic accuracy and remains highly effective in routine diagnostic practice.

In the present study, lung carcinoma was predominantly observed in older individuals, with the highest proportion of cases in the 61-70 years age group (42, 43.3%), followed by 51-60 years (36, 37.1%), and only a small fraction below 40 years. This pattern is consistent with the established epidemiology of lung cancer as a disease of late adulthood related to cumulative carcinogen exposure. Similar age trends have been reported in other institutional studies, including Pujani et al. (mean age of 57 years), Kareliya et al. (peak in 61-70 years; 18, 33.96%), and Aleem et al. (peak in 63-72 years; 19, 34.5%), supporting the reproducibility of this distribution [11,13,14].

A marked male predominance (76, 78.35%) was noted, aligning with most Indian hospital-based series and likely reflecting higher smoking and occupational exposure among men; comparable proportions have been reported by Pujani et al. (91, 71.6%), Kareliya et al. (38, 71.69%), and Aleem et al. (40, 72.7%) [11,13,14]. However, the substantial female representation underscores the increasing burden of lung cancer among women, possibly related to passive smoking, biomass fuel exposure, and air pollution.

Bronchoscopy-guided biopsy was the most common sampling method (72, 74.23%), followed by CT- and USG-guided procedures, reflecting current practice in which bronchoscopic sampling is preferred for central lesions and image-guided techniques are used for peripheral masses [15].

Anatomically, the right lung, particularly the right lower lobe, was the most common site of tumor involvement in the present study, followed by the left upper lobe. A similar right-sided predominance has been reported in prior series, with Pujani et al. documenting the highest proportion of cases in the right lower lobe (36, 28.3%) and Kareliya et al. reporting overall right lung involvement in 29 (54.71%) of cases [11,13]. Although site distribution varies across studies, right lung predominance is frequently observed and may relate to greater lung volume and airflow dynamics. Clinically, malignancy was suspected in the vast majority of patients (94, 96.9%), while only a small proportion were initially considered to have tuberculosis, highlighting the persistent clinicoradiological overlap between lung cancer and granulomatous infectious disease in the Indian setting.

Routine evaluation with H&E-stained sections constituted the primary diagnostic step in the present study, and in many cases, morphology alone was sufficient to suggest the tumor subtype with reasonable confidence. However, in poorly differentiated neoplasms and biopsies lacking definitive architectural or cytological features, morphology was considered insufficient for final classification. Therefore, IHC was performed in all cases to confirm lineage and enable accurate subclassification.

Application of a focused, lineage-directed immunohistochemical panel substantially improved diagnostic precision in the present study. Among squamous markers, p40 demonstrated superior performance, with high sensitivity (93.5%), very high specificity (100%), and overall diagnostic accuracy of 96.7%, whereas p63 showed good sensitivity (92.0%) and specificity (95.2%), but comparatively lower specificity than p40. This difference is well recognized, as p63 may show focal positivity in a subset of adenocarcinomas, a pattern also encountered in a small number of our cases. Similar observations have been reported by Pujani et al., who also found p40 to be a highly sensitive and specific marker for squamous cell carcinoma with excellent diagnostic accuracy [11]. These findings support current recommendations that favor p40 as the preferred squamous marker, particularly in small biopsy specimens where diagnostic certainty and tissue conservation are critical.

For adenocarcinoma, TTF-1 showed excellent sensitivity (96.0%) and high specificity (93.5%), with diagnostic accuracy exceeding 95%, while Napsin-A demonstrated very high specificity (100%) with slightly lower sensitivity (90.9%). The combined use of TTF-1 and Napsin-A, therefore, provides a highly reliable approach for confirming pulmonary adenocarcinoma. Comparable performance has been described by Pujani et al., who reported high sensitivity for TTF-1 (97%) and good specificity for Napsin-A (90%) in adenocarcinoma diagnosis [11]. Neuroendocrine markers synaptophysin and chromogranin demonstrated complete sensitivity and specificity in our series, although this observation should be interpreted cautiously due to the very small number of small cell carcinoma cases included. Likewise, mesothelial markers (WT-1 and calretinin) showed consistent positivity in mesothelioma and absence of staining in other tumor categories. Pan-cytokeratin proved particularly valuable in poorly differentiated and metastatic carcinomas by establishing epithelial origin and guiding subsequent lineage-specific marker application, supporting a stepwise, panel-based diagnostic strategy.

Histopathologically, adenocarcinoma was the most common tumor type (56, 57.7%), followed by squamous cell carcinoma (33, 34.0%), while small cell carcinoma, mesothelioma, metastatic tumors, and poorly differentiated carcinomas constituted a minor proportion. This pattern is consistent with the contemporary global shift toward adenocarcinoma predominance, likely related to changes in smoking behavior, cigarette design, inhalation patterns, and environmental exposures [16,17]. Similar distributions have been reported by Pujani et al., who observed adenocarcinoma in 38 (30%) and squamous cell carcinoma in 32 (25.2%) of cases, and by Kareliya et al., who reported 27 (50.97%) adenocarcinoma and 19 (35.84%) squamous cell carcinoma [11,13]. The relatively lower frequency of small cell carcinoma in biopsy-based series may reflect sampling patterns and referral bias.

Practically, our findings support the use of a limited, high-yield immunohistochemical panel consisting of TTF-1 and Napsin-A for adenocarcinoma and p40 (with or without p63) for squamous cell carcinoma, which provides high diagnostic accuracy while conserving tissue for essential molecular and predictive biomarker testing in small biopsy specimens.

Strengths and limitations

The present study has several important strengths, including systematic correlation of detailed histomorphological assessment with a broad, lineage-directed immunohistochemical panel, and quantitative evaluation of diagnostic performance parameters for individual markers, thereby enhancing the practical and analytical value of the findings for routine diagnostic pathology. The study design reflects real-world small-biopsy reporting conditions, supporting its clinical applicability. However, certain limitations should be considered, including its retrospective, single-center design, which may limit wider generalizability, and the relatively small number of cases in some tumor subgroups, particularly small cell carcinoma and mesothelioma, which may potentially inflate marker performance estimates. In addition, the absence of molecular correlation prevented integrated immunomorphologic-genomic validation of tumor subtypes. Future prospective, multicenter studies with larger sample sizes and molecular correlation would further strengthen and validate these observations.

## Conclusions

This study underscores the pivotal role of careful histomorphological assessment complemented by a focused, lineage-directed immunohistochemical panel, including TTF-1, Napsin-A, p40, and p63, and other lineage-specific markers in achieving accurate subclassification of lung carcinomas on small biopsy specimens. Adenocarcinoma and squamous cell carcinoma constituted the dominant tumor categories in this cohort, reflecting current epidemiological trends. The selected markers showed high diagnostic utility across tumor types, supporting current WHO recommendations that advocate a tissue-conserving, panel-based approach for the subtyping of lung carcinomas while preserving material for molecular and predictive biomarker testing. Overall, a morphology-guided and judiciously applied limited IHC strategy appears highly effective and practical in routine diagnostic workflows, and may facilitate accurate subtype-directed clinical management.
